# Supplementary figures and images for: Comprehensive Immunophenotyping of Monocytes and Dendritic Cells Suggests Distinct Pathophysiology in Chronic Fatigue Syndrome and Long COVID
Source: Int J Mol Sci. 2026 May 17;27(10):4488. doi: 10.3390/ijms27104488 (PMC13206834; doi:10.3390/ijms27104488)

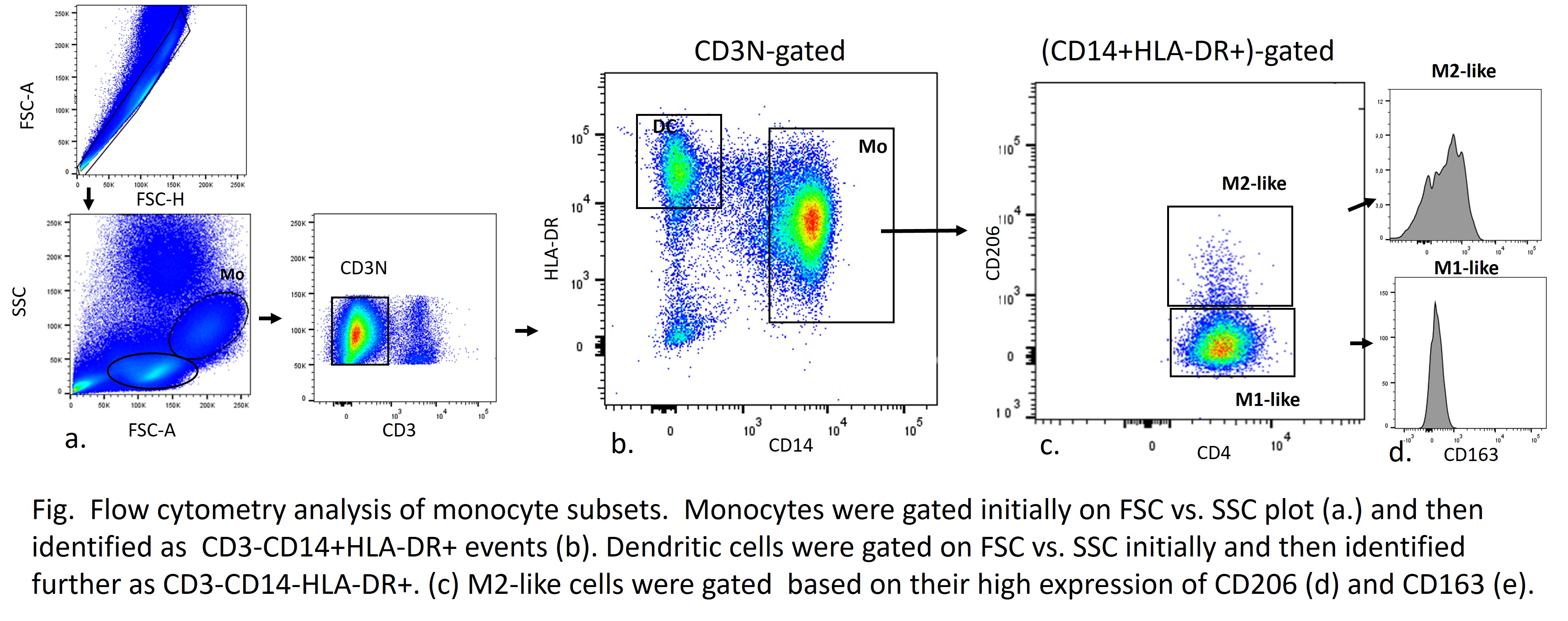

Supplement: Supplementary file 1 [file ijms-27-04488-s001.zip › Figure S1.jpg]
